# A prospective study comparing two methods of pre-hospital triage for trauma

**DOI:** 10.1007/s13304-022-01271-z

**Published:** 2022-03-20

**Authors:** C. Bagnato, K. Ranzato, A. Giarraca, P. Restelli, S. Saronni, G. Gadda, O. Chiara, S. Cimbanassi

**Affiliations:** 1 Acute Care Surgery-Trauma Team, ASST GOM Niguarda, 20162 Milan, Italy; 2 Emergency Department, ASST GOM Niguarda, 20162 Milan, Italy; 3 Department of Medical-Surgical Physiopathology and Transplantation, AcuteCareSurgery-TraumaTeam, ASST GOM Niguarda, 20162 Milan, Italy

**Keywords:** Triage, ACS-COT, TRENAU, Inclusive trauma system, Exclusive trauma system

## Abstract

We conducted a prospective study comparing two different pre-hospital triage tools for trauma: the American College of Surgeons Committee on Trauma (ACS-COT) field triage decision scheme and the TRENAU score. The main objective was to evaluate which triage tool was more appropriate in the setting of Lombardy's trauma system. Data were collected from the population of trauma patients admitted to Niguarda hospital in Milan from January to June 2021. RStudio and Excel were used for data analysis. For each triage tool performance measures, Receiver Operating Characteristics (ROC) curves, and overtriage and undertriage rates were obtained. A total of 1439 injured patients admitted through 118 pre-hospital Emergency Medical Services (EMS) were included in the study. The ACS-COT triage tool showed a good accuracy but an excessive overtriage rate (59%). The TRENAU triage tool had a moderately good accuracy and a low overtriage rate (23%) while maintaining an acceptable undertriage rate (3.9%). The TRENAU triage tool proved to be efficient in optimizing the use of resources dedicated to trauma care while resulting safe for the injured patient. In a modern trauma system such as Lombardy's it would be more appropriate to adopt the TRENAU score over the ACS-COT field triage decision scheme.

## Introduction

Trauma is a complex and still largely neglected disease, responsible for about 5.8 million deaths annually around the world [1]. Trauma has also a high social impact, being the major cause of death and disability among the population under 35 years old. The most frequent mechanism of injury (MOI) are road accidents (29%), followed by falls (12.6%) and assault (9.16%) [2].

To optimize trauma care and thus minimize the number of preventable deaths, trauma related disability and resource wasting, Western Countries have developed Trauma Systems within their national health systems. Within the Trauma System various phases of trauma care^1^ are organized and embedded according to a well-structured protocol.

Injured patients are treated in trauma centers, which can supply different level of trauma care. In Italy there are three types of trauma centers: *Centro Trauma ad Alta Specializzazione* (CTS, a highly specialized trauma center), *Centro Trauma di Zona* (CTZ, a local trauma center) and *Presidio di Stabilizzazione per Traumi* (PST, a hospital unit for trauma stabilization). A CTS manages the most severely injured patients, providing the highest level of care. A CTS is equivalent to a level 1 trauma center and has a catchment area of about 2 million inhabitants. A CTZ, which corresponds to a level 2 trauma center, manages major trauma except for cases that require high level of professional expertise. A PST, similarly to a level 3 trauma center, takes care of minor trauma or stabilizes critically injured patients before transferring them to a higher level trauma center [3].

Regarding the distribution of trauma centers on the territory, two models of trauma systems can be distinguished: an exclusive trauma system and an inclusive trauma system. In an exclusive trauma system acute care facilities and trauma centers are two separated entities. In this type of system the Emergency Medical Services (EMS) should therefore centralize all trauma patients to trauma centers. In an inclusive system the territory is served by several acute care facilities, the majority of which is also equipped with a trauma center. Each trauma center can offer different levels of care depending on the necessities of every geographical area. In such a model, the task of the EMS’ personnel is to estimate the severity of injured patients based on pre-determined triage criteria. Patients are thus transported to the hospital capable of providing the adequate level of care in a timely fashion [4].

To optimize the pre-hospital phase of trauma care, a trauma system should adopt triage tools that better adapt to the local environment. In the U.S.A. paramedics implement the American College of Surgeons Committee on Trauma (ACS-COT) field triage decision scheme: four successive steps, each consisting of a specific set of criteria, are followed to evaluate the injured patient [5, 6]. Italy has also been adopting the ACS-COT triage tool to evaluate the trauma patient on the scene of the event. The new 2020 guidelines however suggest the use of the triage score developed in the context of the Northern French Alps (TRENAU) trauma system. An effort is thus being made to promote the transition from the ACS-COT triage tool to the TRENAU triage tool where appropriate. The parameters assessed by the TRENAU criteria are similar to those assessed by the ACS-COT field triage decision scheme, with the addition of the response of the patient to treatment. Response to treatment can be evaluated during the pre-hospital phase since in France, as well as in Italy, the EMS personnel is also composed of nurses and physicians [7, 8]. The TRENAU grading system establishes three levels of clinical severity: class A (unstable patient despite treatment), B (stabilized after treatment or anatomic criteria) or C (stable with high energy MOI, age and comorbidities) [1].

The present study aims to compare the triage tool developed by the ACS-COT and the TRENAU score to establish which one is better suited to the regional setting of Lombardy’s trauma system.

## Methods

### Study design and setting

A prospective observational cohort study was conducted between January 23rd and June 24th 2021.

Trauma patients were admitted to the Emergency Department (ED) of ASST Grande Ospedale Metropolitano Niguarda, a CTS for the metropolitan area of Milan. For all suspected major trauma patients a multidisciplinary Trauma Team (TT) was activated at the EMS’ notice. Niguarda’s TT consists of general surgeons, anesthesiologists, radiologists, trauma nurses, radiology’s technicians and orderlies [9].

### Data collection

Data were collected from pre-hospital EMS’ reports, dispatch on major trauma papers, emergency room reports and medical charts and were organized in a spreadsheet file.

The following information was included in the database: age, gender, EMS type (basic life support, intermediate life support, advanced life support or helicopter rescue), notice by the 118 dispatch center based on the ACS-COT triage criteria, trauma type (closed vs open/piercing), event type (single or multiple, self-inflicted or inflicted by a third party), trauma mechanism (road accident, fall, assault, sport injury, animal related injury or other), ACS-COT triage criteria, risk factors and comorbidities, physiologic parameters on the scene, outcome (discharge by the ED, admission to the operating theater, admission to the intensive care unit, damage control maneuvers performed in the emergency room, death in the ED, hospitalization), Injury Severity Score (ISS), TT activation, TRENAU score, New Trauma Score for Triage (T-NTS).

In this study EMS personnel assessed injured patients on the scene using the ACS-COT field triage decision scheme. If the ACS-COT criteria for major trauma were met the 118 dispatch center notified the ED where the TT was activated in advance and the triage nurse filled in the dispatch on major trauma papers with information regarding the traumatic event and the patient's characteristics.

The TT was considered as correctly activated when the triage criteria effectively identified major trauma. Major trauma was defined by having an ISS higher than 15 and/or the Need For Trauma Intervention (NFTI+). The ISS was calculated considering the Abbreviated Injury Score (AIS) 2015 catalogue [10, 11]. The NFTI indirectly describes the impact of the traumatic event on the patient through the intensity of the therapeutic maneuvers needed to replenish the body's physiological reserves. The therapeutic maneuvers considered in the study were: damage control resuscitation in the ED, direct discharge to the operating room or the intensive care unit from the ED, death of the patient in the ED [12].

Afterward, each patient was graded using the TRENAU score based on the information received by the EMS. The correct activation of the TT was assessed simulating a situation in which the EMS implemented the TRENAU triage tool instead of the ACS-COT field triage decision scheme on the scene of the traumatic event. A patient who met the criteria for class A and B was considered in need of a TT activation at a CTS. The TRENAU score was considered appropriate if it would have resulted in the activation of the TT for major trauma patients (ISS higher than 15 and/or NFTI+). Patients who met the criteria for class C would have been transported to a CTZ had the TRENAU grading system been used on the scene of the event instead of the ACS-COT triage tool. Patients who did not meet any criteria would have been considered mildly injured and thus transported at the nearest hospital.

### Statistical analysis

Data were analyzed using Excel© version 16.43, RCRAN© version 4.1.1 and RStudio Desktop© version 1.4.1717.

The ACS-COT triage tool and the TRENAU triage tool were compared through performance measures with 95% Confidence Intervals (CIs). In particular, the focus was on sensitivity and specificity which express the capability of the triage tool to discriminate between major and minor trauma.

Receiver Operating Characteristics (ROC) curves with 95% confidence bands were plotted for each triage tool. The Area Under the Curve (AUC) of the ROC curve depicts the accuracy of the triage tool and can range from 0.5 to 1. The ideal triage tool which can perfectly differentiate major trauma from minor trauma would have an AUC of one. An AUC lower than 0.7 defines a poor triage tool, between 0.71 and 0.8 defines a moderately good triage tool and higher than 0.81 defines a good triage tool. Plotting ROC curves allows to immediately visualize performance information about the ACS-COT field triage decision scheme and the TRENAU score and promptly compare their accuracy.

Overtriage and undertriage rates (referred to the centralization of major trauma at a CTS) with 95% CIs were obtained using the Cribari Matrix method. 95% CIs were calculated as:

$$p\pm 1.96\times \sqrt{\frac{p\times (1-p)}{n}}$$

where *p* is the proportion of patients that were undertriaged or overtriaged while *n* is the total number of patients with major trauma or who matched with at least one of the triage criteria considered for major trauma. Overtriage is defined as the rate of mild injuries misidentified as major trauma by the triage tool. Undertriage is defined as the rate of severely injured patients that the triage tool missed to identify and were thus transported to an under-qualified trauma center or received late treatment. When designing a triage tool it is important to control the rates of overtriage and undertriage. In particular the undertriage rate should be kept to a minimum since missing to identify major trauma can jeopardize the patient's safety resulting in higher rates of preventable deaths and worsening prognosis. A high overtriage rate does not endanger the patient’s life in the near future, however it should be contained as it entails a waste of human and financial resources that could lead to the system’s collapse in the long term. The ACS-COT suggests maintaining the undertriage rate at less than 5% and the overtriage rate between 25 and 35% [6]. This aspect should be considered to determine which triage tool is more appropriate in a specific trauma system.

## Results

A total of 4909 trauma patients (excluding those who presented burns as the only MOI) were admitted to Niguarda’s ED during the period of the study. Only the 1439 patients who were transported by EMS were included in the study. The study population characteristics are summarized in Table 1.

**Table 1:** Study sample population’s descriptive table

	N° Tot (%)	N° Major Trauma	ISS	N° Minor Trauma	ISS	*P *-value
			Median (IQR)		Median (IQR)	
Age						0.176
1–5	71 (5)	1	18 (0)	70	1 (0)	0.464
6–18	170 (12)	15	18 (14–25)	155	1 (1–4)	0.2732
19–65	780 (54)	105	19 (16–27)	675	2 (1–4)	0.884
66–80	167 (12)	13	25 (21–26)	154	2 (1–5)	0.185
81–97	251 (17)	9	25 (25–48)	242	4 (1–5)	0.130
Gender						0.000515*
Male	897 (62)	113	20 (16–27)	784	2 (1–4)	
Female	542 (38)	30	22 (16–25)	512	2 (1–4)	
EMS type						<2e^−16*^
BLS	1136 (79)	17	17 (16–22)	1119	1 (1–4)	
ILS	3	0		3	1 (1–2)	
ALS	252 (18)	99	21 (16–27)	153	4 (1–9)	
Helicopter	48 (3)	27	21 (17–34)	21	8 (5–9)	
Trauma type						0.718
Closed	1085 (75)	107	21 (16–26)	978	2 (1–4)	
Open/piercing	354 (35)	36	18 (15–29)	318	2 (1–4)	
Event type						0.000372*
Single	1343 (93)	122	20 (16–26)	1221	2 (1–4)	
Multiple	96 (7)	21	21 (17–29)	75	1 (1–4)	
Trauma dynamic						0.305
Road accidents	543 (38)	86	18 (16–25)	457	2 (1–4)	
Falls	591 (41)	32	25 (19–29)	559	2 (1–4)	0.3856
Assault	137 (10)	3	17 (17–46)	588	1 (1–4)	<2e^−16*^
Sport injuries	10 (1)	0		10	1 (0)	
Animals	18 (1)	0		18	1 (1–2)	
Wounds	76 (5)	10	25 (12–33)	66	1 (1–4)	1
Crushing	18 (1)	7	18 (12–24)	11	1 (1–2)	0.0297 *
Other	46 (3)	5	19 (18–25)	41	1 (1–2)	
ACS-COT criteria						<2e^−16*^
No notice	1115 (77)	10	18 (17–25)	1105	1 (1–4)	
Notice	324 (23)	133	20 (16–27)	191	4 (1–9)	
TRENAU criteria						<2e^−16*^
A + B	118 (8)	91	25 (17–34)	27	4 (2–7)	
C + NI	1321 (92)	52	17 (13–21)	1269	2 (1–4)	
T-NTS						3.78 e^−11*^
>17	740 (96)	52	18 (15–23)	688	1 (1–4)	
≤ 17	27 (4)	21	27 (19–34)	6	7 (4–10)	
ISS						<2e^−16*^
>15	112 (8)	112	25 (18-31)	0	2 (1–4)	
≤15	1327 (92)	31	9 (7-11)	1296		

Values marked with a * are statistically significant. A level of significance of 0.05 was applied for all tests

*IQR  * Interquartile Range, *BLS* Basic Life Support, *ILS* Intermediate Life Support, *ALS* Advanced Life Support, *NI* = Not Included

Of all the 1439 patients included in the study, 143 met the criteria for major trauma (ISS greater than 15 and/or NFTI+). The ED was notified by the EMS in 22.51% (324/1439) of cases, 41.04% of whom (133/324) were confirmed being major trauma (Table 2). Employing the TRENAU grading system, 118 patients (of which 91 were major traumas) were classified as grade A or grade B, 488 (of which 43 were major traumas) as grade C and 833 (of which 9 were major traumas) as "not included" (Table 3).

**Table 2:** EMS notice and trauma severity

	*N*	ISS Median (IQR)
Access through EMS	1439	2 (1–5)
Notice	324	9 (4–18)
Major trauma	133	20 (16–27)
Minor trauma	191	4 (1–9)
No notice	1115	1 (1–4)
Major trauma	10	18 (17–25)
Minor trauma	1105	1 (1–4)

*IQR *Interquartile Range

**Table 3:** TRENAU classes and trauma severity

	*N*	ISS
		Median (IQR)
GRADE A	20	34 (25–75)
Major trauma	19	34 (25–75)
Minor trauma	1	3 (0)
GRADE B	98	17 (9–25)
Major trauma	72	22 (16–28)
Minor trauma	26	4 (2–8)
GRADE C	488	4 (1–8)
Major trauma	43	17 (13–22)
Minor trauma	445	4 (1–5)
NOT INCLUDED	833	1 (1–4)
Major trauma	9	18 (16–19)
Minor trauma	824	1 (1–4)

*IQR * Interquartile range

The performance measures of the ACS-COT triage tool and the TRENAU score were compared (Tables 4 and 5) and statistically significant differences were found. In particular, the sensitivity of the TRENAU triage tool was notably lower than that of the ACS-COT criteria: 61% (95%CI of 52–69%) against 93% (95%CI of 87–97%). This resulted however in a specificity of almost 100% (98% with a 95%CI of 97–99%) against that of the ACS-COT triage tool which amounted only up to 85% (95%CI of 83–87%).

**Table 4:** Performance measures of the ACS-COT criteria with 95% CIs

	Value	95% CI
Apparent prevalence	0.22	(0.20, 0.25)
True prevalence	0.10	(0.08, 0.12)
Sensitivity	0.93	(0.87, 0.97)
Specificity	0.85	(0.83, 0.87)
Positive predictive value	0.41	(0.35, 0.46)
Negative predictive value	0.99	(0.98, 1.00)
Positive likelihood ratio	6.31	(5.50, 7.25)
Negative likelihood ratio	0.08	(0.05, 0.15)

**Table 5:** Performance measures of the TRENAU triage tool with 95% CIs

	Value	95% CI
Apparent prevalence	0.08	(0.07, 0.09)
True prevalence	0.10	(0.08, 0.12)
Sensitivity	0.61	(0.52, 0.69)
Specificity	0.98	(0.97, 0.99)
Positive predictive value	0.76	(0.67, 0.84)
Negative predictive value	0.96	(0.95, 0.97)
Positive likelihood ratio	29.09	(19.58, 43.23)
Negative likelihood ratio	0.40	(0.33, 0.49)

**Fig. 1:**
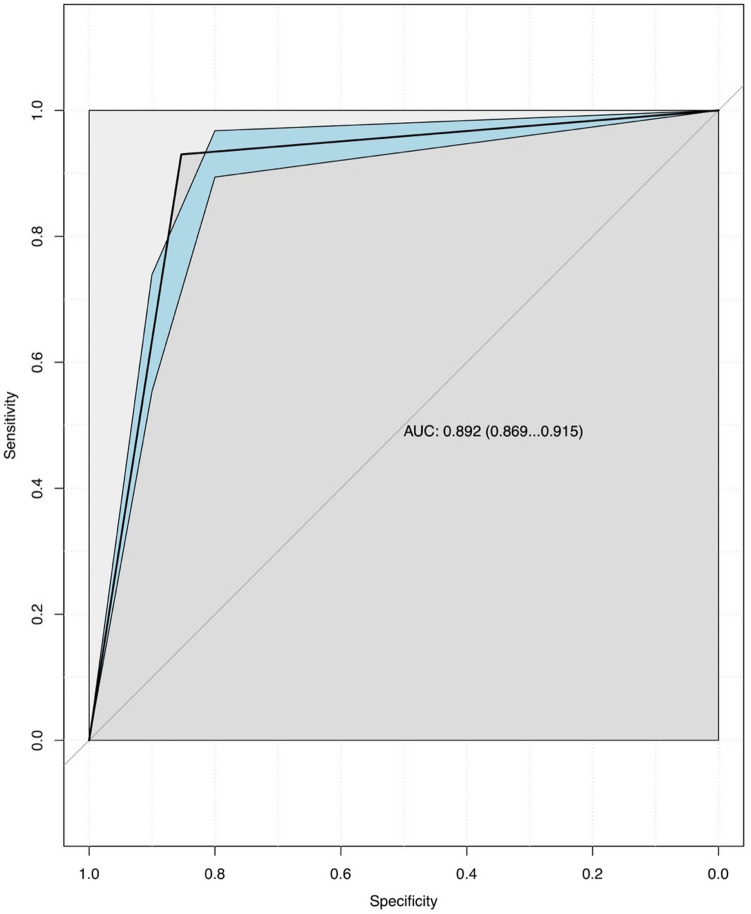
ACS-COT triage tool’s ROC curve with 95% CIs

**Fig. 2:**
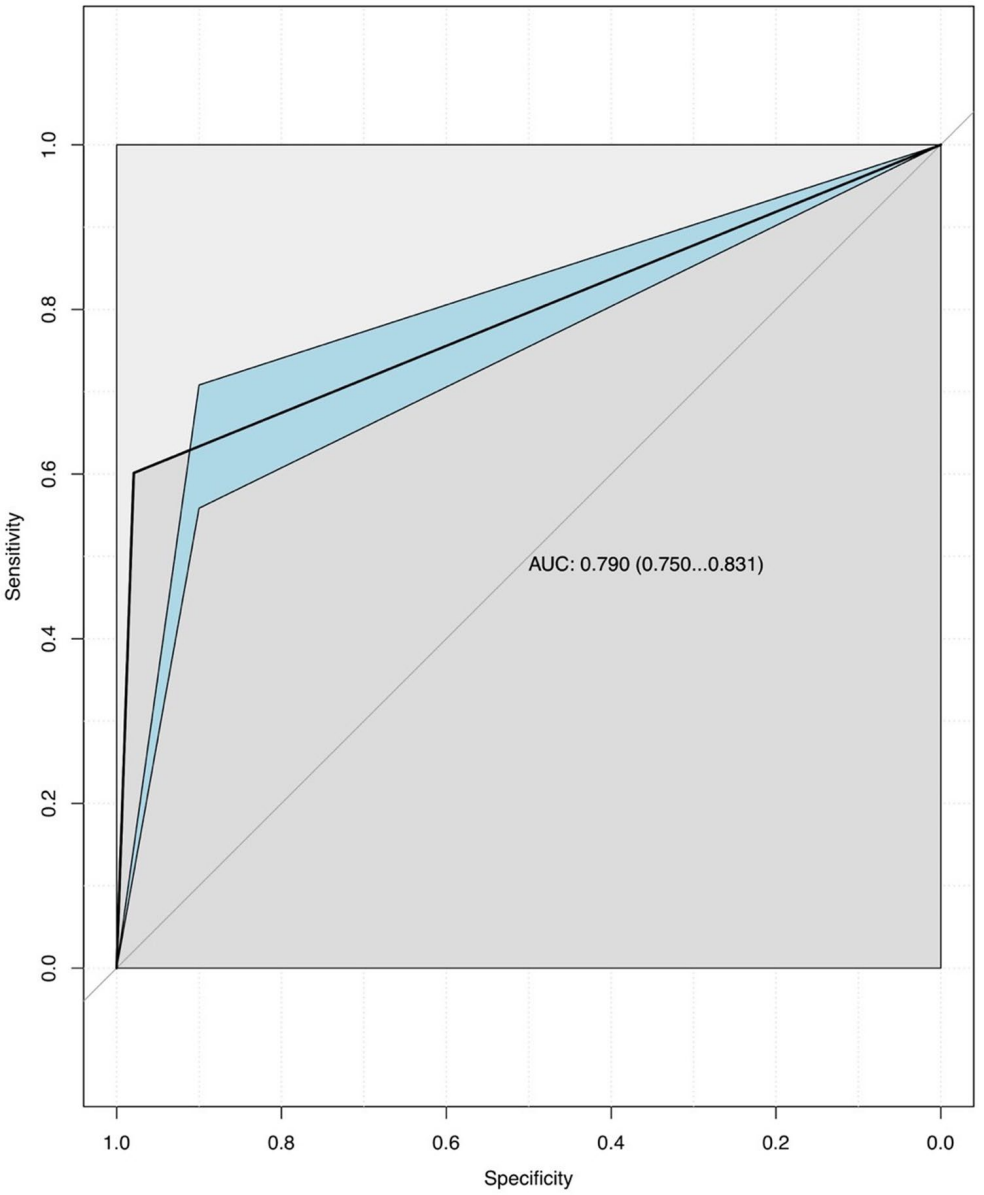
TRENAU triage tool’s ROC curve with 95% CIs

The AUC of the ROC curves depicted two different levels of accuracy for the ACS-COT triage tool and the TRENAU triage tool (Figs. 1 and 2). The difference between the ACS-COT triage criteria and the TRENAU criteria’s accuracy is statistically significant: the former is more accurate (AUC of 0.892 with CI of 0.869–0.915), however the latter can be still considered a moderately good test (AUC of 0.790 with CI of 0.750–0.831).

There was a statistically significant difference also in overtriage and under- triage rates as shown in Table 6. Overtriage was significantly lower when implementing the TRENAU triage tool rather than the ACS-COT field triage decision scheme: 23% (95%CI of 15.4–30.6%) against 59% (95%CI of 53.6–64.4%). The undertriage rate of the TRENAU score still maintained under the 5% limit suggested by the ACS-COT [6] at 3.9% (95% CI of 2.9–4.9%).

**Table 6:** Overtriage and undertriage rates

	ACS-COT triage tool	TRENAU triage tool
Overtriage %	59%	23%
N° (95% ICs)	0.59 (0.536–0.644)	0.23 (0.154–0.306)
Undertriage %	1%	3.9%
N° (95% ICs)	0.01 (0.004–0.016)	0.039 (0.029–0.049)

## Discussion

Considering the results as a whole and referring them to the setting in which they were obtained, it can be observed how the ACS-COT field triage decision scheme is characteristic of a trauma system in its early stage. A triage tool such as the one developed by the ACS-COT complies with the requirements of a trauma system which starts as exclusive or is either still developing an organized network of trauma centers and a proper personnel expertise. Sensitivity has to be high to minimize the number of false negatives (major trauma which is identified as minor by the triage criteria, i.e. the undertriaged patients). Even if it means having an excessive overtriage rate. In a young trauma system undertriaged patients would not receive the appropriate level of care, being transported to facilities not included in the trauma system or which are still developing and do not have the adequate expertise. In other words, EMS within a trauma system at its early stage should adopt a triage tool that works as a screening test, reducing the false negatives to a minimum and ruling in all the possibly diseased patients. The ACS-COT field triage decision scheme was in fact appropriate in Lombardy about 20 years ago, when Italy started to build a network of organized trauma systems at a regional level. The ACS-COT triage tool has in fact a very high sensitivity (93%) and accuracy (89.2%) which define a minimum rate of undertriage (1%). This however results in a lower specificity (85%) and thus a much higher rate of overtriage (59%) which greatly exceeds the threshold suggested by the ACS-COT.

On the contrary, a triage tool like the TRENAU score better suits a trauma system such as Lombardy’s at the present time: inclusive, which had the time to appropriately develop its network of trauma centers and strengthen its personnel expertise. The TRENAU triage tool has a lower sensitivity (61%) and accuracy (79%) which nonetheless define a still acceptable rate of undertriage (3.9%) according to the ACS-COT. On the other hand, this results in a very high specificity (98%) and thus a much lower overtriage rate (23%) which is within the threshold set by the ACS-COT. This way, overcrowding of trauma centers would be prevented, resource wasting would be minimized, and patient care improved. Optimizing trauma care is essential nowadays. It is not sustainable to accept high overtriage rates anymore as European health care systems are struggling to maintain their status as free and universal [13]. A high specificity is typical of a diagnostic test, which is supposed to rule out all the patients that do not have the disease and identify only the true positives. This clearly results in a higher number of false negatives due to the decrease in sensitivity, which should nonetheless always be kept under an acceptable threshold. Since the TRENAU triage tool works as a diagnostic test rather than a screening test, it allows to select and centralize to the CTS mainly the true major trauma.

Furthermore, in a trauma system with different levels of care, undertriaged patients would not be neglected but treated in a less specialized trauma center with an adequate expertise, namely a CTZ. It should also be noted that the great majority of Lombardy’s CTZs are equipped with a neurosurgical unit, which is essential as the majority of trauma patients present with traumatic brain injuries [14]. Moreover, in a modern trauma system, a triage tool should also be able to identify patients potentially at risk of an unfavorable outcome, even when they are not labelled as major trauma by the triage criteria. They could either be undertriaged patients or patients who are more prone to evolve unfavorably rather than get better and should therefore be directed towards a CTZ. In favor of this statement, it should be pointed out that in this study the majority of the patients undertriaged with the TRENAU score were classified as grade C (43 out of 52) and would therefore have been treated in a CTZ, guaranteeing them an appropriate level of care.

Even though criteria based solely on trauma dynamic and patient's characteristics seem to be the most relevant cause of overtriage [15, 16], it is important not to underestimate the condition of grade C patients since age and MOI showed to correlate with injury severity [6, 17, 18]. Some studies even suggested the need for specialized geriatric trauma teams and specific triage criteria for elderly patients and children [19–21].

Only two other studies that evaluated the efficacy of the TRENAU grading system and compared it with other pre-hospital triage tools were found in literature.

The TRENAU score is in fact fairly new since it was implemented only in 2015 by Bouzat et al. [1]. In his study Bouzat compared a graded group of patients with a non-graded group of patients within the Northern French Alps trauma system. The implementation of the TRENAU score appeared more appropriate in a system involving physicians in the pre-hospital phase opposed to the ACS-COT triage tool for paramedics. Considering the graded group, sensitivity showed to be higher (92%) while specificity was much lower (41%) compared to the current study. In addition, undertriage and overtriage rates exceeded the threshold suggested by the ACS. The difference in results could be explained by the lack in experience of the French EMS in implementing the pre-hospital triage tool in 2015 and by the dissimilar geographical area in which the two studies took place (the Northern French Alps are a mountainous region while Milan is a plain metropolitan area) [22].

A more recent and comprehensive study regarding the TRENAU classification is a systematic review with meta-analysis conducted by Gianola et al.[19]*.* which compares the TRENAU score with other triage tools (e.g. the ACS-COT field triage decision scheme, Vittel Triage Criteria, New Trauma Score, etc.) according to their ROC curve and net clinical benefit. The TRENAU score, together with the NTS, appeared to be the most accurate triage tool in the adult population.

This study has a few limitations. Data were collected in a single level 1 trauma center and reflect the population of a highly urbanized area. The study was conducted during the COVID-19 pandemic and the restrictions introduced by the government had an impact on the epidemiology of trauma and the organization of the healthcare system [23].

## Conclusion

When comparing different triage tools, the local setting in which they are implemented should be considered. A young trauma system benefits from a highly sensitive triage tool like the ACS-COT field triage decision scheme. A mature trauma system such as Lombardy’s, which has developed a functioning and up-to-date trauma network with different levels of trauma centers, should adapt its triage tool accordingly. In this kind of setting, the TRENAU score proved to be extremely effective in reducing overtriage without compromising the patient’s safety. As such, Lombardy is encouraged to follow the new Italian guidelines in adopting the TRENAU score as a pre-hospital triage tool. This however could differ for other Italian regions where trauma systems might not be up to standard yet and the adoption of a new triage tool should thus be considered case-by-case.

## Footnotes

1 Trauma prevention, pre-hospital care, acute in-hospital trauma care, post-hospital care, research activities and education.

## References

[1] Bouzat P, Ageron F, Brun J (2015). A regional trauma system to optimize the pre-hospital triage of trauma patients. Crit Care.

[2] Alberdi F, Garcia I, Atutxa L (2014). Epidemiology of severe trauma. Med Intensiva.

[3] Chiara O, Gordini G, Nardi G, et al. (2012) Trauma Update. La cura definitiva del trauma maggiore. Elsevier Italia, Milano

[4] Kozar RA, Sanddal ND (2016). Role of level iv trauma centers in an inclusive trauma system: the injured elderly. Am J Surg.

[5] Sasser SM, Hunt RC, Faul M (2011). (2012) Guidelines for field triage of injured patients: recommendations of the national expert panel on field triage. Morb Mortal Wkly Rep.

[6] Rotondo M, Cribari C, Smith R (2014). Resources for optimal care of the injured patient.

[7] Centro Nazionale per l’eccellenza clinica la qualità e la sicurezza delle cure (June 2020) Raccomandazioni 1-4 della linea guida sulla gestione integrata del trauma maggiore dalla scena dell’evento alla cura definitiva. LG-ISS-SNLG-3

[8] David JS, Bouzat P, Raux M (2019). Evolution and organisation of trauma systems. Anaesth Crit Care Pain Med.

[9] Chiara O, Cimbanassi S (2008) Protocolli per la gestione intraospedaliera del trauma maggiore. Elsevier srl

[10] Baker SP, Neill B, Haddon Jr W, et al (1974) The injury severity score: a method for describing patients with multiple injuries and evaluating emergency care. J Trauma Acute Care Surg 14(3):187–196 4814394

[11] Bolorunduro OB, Villegas C, Oyetunji TA (2011). Validating the injury severity score (iss) in different populations: Iss predicts mortality better among hispanics and females. J Surg Res.

[12] Roden-Foreman JW, Rapier NR, Foreman ML (2019). Rethinking the definition of major trauma: the need for trauma intervention outperforms injury severity score and revised trauma score in 38 adult and pediatric trauma centers. J Trauma Acute Care Surg.

[13] Lega F (2020). Economia e management sanitario: Settore, sistema, aziende, protagonisti.

[14] Chieregato A, Volpi A, Gordini G, et al (2017) How health service delivery guides the allocation of major trauma patients in the intensive care units of the inclusive (hub and spoke) trauma system of the emilia romagna region (Italy) a cross-sectional study. BMJ Od trauma team activation protocol: apen 7(9):e016415 10.1136/bmjopen-2017-016415 PMC5640142 28965094

[15] Boyle MJ (2007). Is mechanism of injury alone in the prehospital setting a predictor of major trauma–a review of the literature. J Trauma Manag Outcomes.

[16] Uleberg O, Vinjevoll O, Eriksson U (2007). Overtriage in trauma– what are the causes?. Acta Anaesthesiol Scand.

[17] Bardes JM, Benjamin E, Schellenberg M (2019). Old age with a traumatic mechanism of injury should be a trauma team activation criterion. J Emerg Med.

[18] Recicar J, Barczyk A, Duzinski S (2016). Does restraint status in motor vehicle crash with rollover predict the need for trauma team presence on arrival? An atomac study. J Pediatr Surg.

[19] Gianola S, Castellini G, Biffi A (2021). Accuracy of pre-hospital triage tools for major trauma: a systematic review with meta-analysis and net clinical benefit. World J Emerg Surg.

[20] Hung KK, Yeung JH, Cheung CS, et al (2019) Trauma team activation criteria and outcomes of geriatric trauma: 10 year single centre cohort study. Am J Emerg Med 37(3):450–456 10.1016/j.ajem.2018.06.011 30041911

[21] Thorsen K, Narvestad JK, Tjosevik KE, et al (2021) Changing from a two-tiered to a one-tiered trauma team activation protocol: a before– after observational cohort study investigating the clinical impact of undertriage. Eur J Trauma Emerg Surg. 10.1007/s00068-021-01696-y. 10.1007/s00068-021-01696-y PMC9532293 34023928

[22] Girard E, Jegousso Q, Boussat B (2019). Preventable deaths in a French regional trauma system: a six-year analysis of severe trauma mortality. J Visc Surg.

[23] Giudici R, Lancioni A, Gay H (2021). Impact of the COVID-19 outbreak on severe trauma trends and healthcare system reassessment in lombardia, Italy: an analysis from the regional trauma registry. World J Emerg Surg.

